# Case Report: A novel mutation in *WFS1* gene (c.1756G>A p.A586T) is responsible for early clinical features of cognitive impairment and recurrent ischemic stroke

**DOI:** 10.3389/fgene.2023.1072978

**Published:** 2023-02-02

**Authors:** Yuan Chen, Miao Zhang, Yuying Zhou, Pan Li

**Affiliations:** ^1^ Department of Neurology, Tianjin Huanhu Hospital, Clinical College of Neurology, Neurosurgery, and Neurorehabilitation, Tianjin Medical University, Tianjin, China; ^2^ Department of Neurology, Tianjin Huanhu Hospital affiliated to Nankai University, Tianjin University Huanhu Hospital, Tianjin, China; ^3^ Tianjin Key Laboratory of Cerebral Vascular and Neurodegenerative Diseases, Tianjin Neurosurgery Institute, Tianjin Huanhu Hospital, Tianjin, China

**Keywords:** *WFS1* gene mutation, wolframin protein, cognitive impairment (CI), ischemic cerebral infarction, neurodegeneration

## Abstract

Wolfram syndrome 1 (*WFS1*) gene mutations can be dominantly or recessively inherited, and the onset of the clinical picture is highly heterogeneity in both appearance and degree of severity. Different types of *WFS1* mutations have been identified. Autosomal recessive mutations in the *WFS1* gene will underlie Wolfram syndrome 1 (WS1), a rare and severe neurodegenerative disease characterized by diabetes insipidus, diabetes mellitus, optic atrophy, deafness, and other neurological, urological and psychiatric abnormalities. Other WFS1-related disorders such as low-frequency sensorineural hearing impairment (LFSNHI) and Wolfram syndrome-like disease with autosomal dominant transmission have been described. It is difficult to establish genotype-phenotype correlations because of the molecular complexity of wolframin protein. In this report, we presented a case of *WSF1* gene mutation-related disease with cognitive impairment as the initial symptom and recurrent cerebral infarction in the course of the disease. Brain structural imaging results suggested decreased intracranial volume, dramatically reduced in cerebral cortex and cerebellum regions. Multimodal molecular imaging results suggested Tau protein deposition in the corresponding brain regions without Aβ pathology changes. These pathological changes may indicate a role of *WFS1* in neuronal vulnerability to tau pathology associated with neurodegeneration and ischemia-induced damage.

## 1 Introduction

Wolfram syndrome 1 (*WFS1*) has been identified as the causative genes for Wolfram Syndrome type 1 (WS1) (Inoue et al., 1998; Amr et al., 2007). *WFS1* mutations may be dominantly or recessively inherited, and the onset of the clinical symptoms vary widely in both appearance and severity (Rigoli et al., 2018). Autosomal recessive mutations in the *WFS1* gene will cause the classical form of WS1, characterized by diabetes insipidus, diabetes mellitus, optic atrophy and deafness. However, several other WS1 clinical phenotypes associated with dominant inheritance of the *WFS1* gene have been identified (Rigoli et al., 2018). Patients with a autosomal dominant mutation pattern are more likely to present with Wolfram syndrome-like disease, which is characterized by a wide spectrum of neurological and psychiatric manifestations, including brain stem and cerebellum atrophy, seizure, depression, anxiety, and cognitive impairment (Rando et al., 1992; Barrett et al., 1995; Scolding et al., 1996; Eiberg et al., 2006; Lipson et al., 2006; Chaussenot et al., 2011; Rendtorff et al., 2011). So it is difficult to establish the correlations between the genotype and phenotype, because of the molecular complexity and genetic variability of WS1, as well as the different clinical features and the small size of patient cohorts (Rigoli et al., 2018).

The *WFS1* gene was identified and maps to chromosome 4p16, and consists of eight exons spanning 33.4 kb of genomic DNA (Inoue et al., 1998; Strom et al., 1998). Currently, more than 200 distinct mutations have been identified in *WFS1* gene. Majority of these are located in the largest exon 8 of *WFS1* gene (Yu et al., 2010), which encodes for the transmembrane region and the carboxy terminal of wolframin protein at the luminal site of the endoplasmic reticulum (ER) membrane (Rohayem et al., 2011). As an ER-resident protein, wolframin protein, which is highly distributed in the brain and the pancreas, has an important role in maintaining the Ca^2+^ homeostasis of ER and regulating the ER stress-induced unfolded protein response (UPR) in different cell populations (Li et al., 2020). Some recent studies have clearly revealed the role of wolframin protein in the communication between endoplasmic reticulum and mitochondria. It was showed that wolframin protein forms a complex with neuronal calcium sensor 1 (NCS1) and the inositol 1,4,5-trisphosphate receptor (IP_3_R) to facilitate Ca^2+^ transfer between the ER and mitochondria. In fibroblasts from WFS1-deficient patients, NCS1 abundance was reduced, along with reduced ER-mitochondrial interactions and Ca^2+^ exchange; in contrast, NCS1 overexpression not only restored ER-mitochondria interactions and Ca^2+^ transfer, but also rescued mitochondrial dysfunction. Wolframin protein, NCS1, and IP_3_R may be part of a complex associated with ER-mitochondria contact sites. In healthy cells, wolframin protein interacts with NCS1 and may prevent its degradation. When WFS1 is lost, the WFS1/NCS1/IP3R complex is disrupted and NCS1 is partially degraded or destabilized. Consequently, defective calcium influx within mitochondria leads to mitochondrial bioenergetic dysfunction, which may result in the activation of cell death in the tissues affected by WS1 (Angebault et al., 2018). This greatly contributes to clarify that mitochondrial dysfunction is not a primary event in the pathogenic mechanism of WS1, but may occur secondarily as a byproduct of calcium mishandling (La Morgia et al., 2020). In addition, different types of *WFS1* mutations have been identified, and have been shown to exert different effects on wolframin function. Individuals carrying mutations with a predicted complete loss of function will exhibit a severe and typical phenotypes of WS1, however, patients carrying mutations with a putative minor loss of function, possibly tend to present with Wolfram syndrome-like disease with incomplete clinical manifestation (Rohayem et al., 2011; Rigoli et al., 2018).

In this report, we reported the clinical and neuroimaging observations in a patient with cognitive impairment and recurrent ischemic stroke. Although, the clinical features of this patient are insufficient to meet the diagnostic criteria for WS1, the novel missense mutation in exon 8 identified by *WFS1* (c.1756G>A p.A586T) gene sequencing may serve as an etiological basis for explaining the neurological and psychiatric symptoms, and neuroimaging changes.

## 2 Case report

### 2.1 Clinical history

**FIGURE 1:**
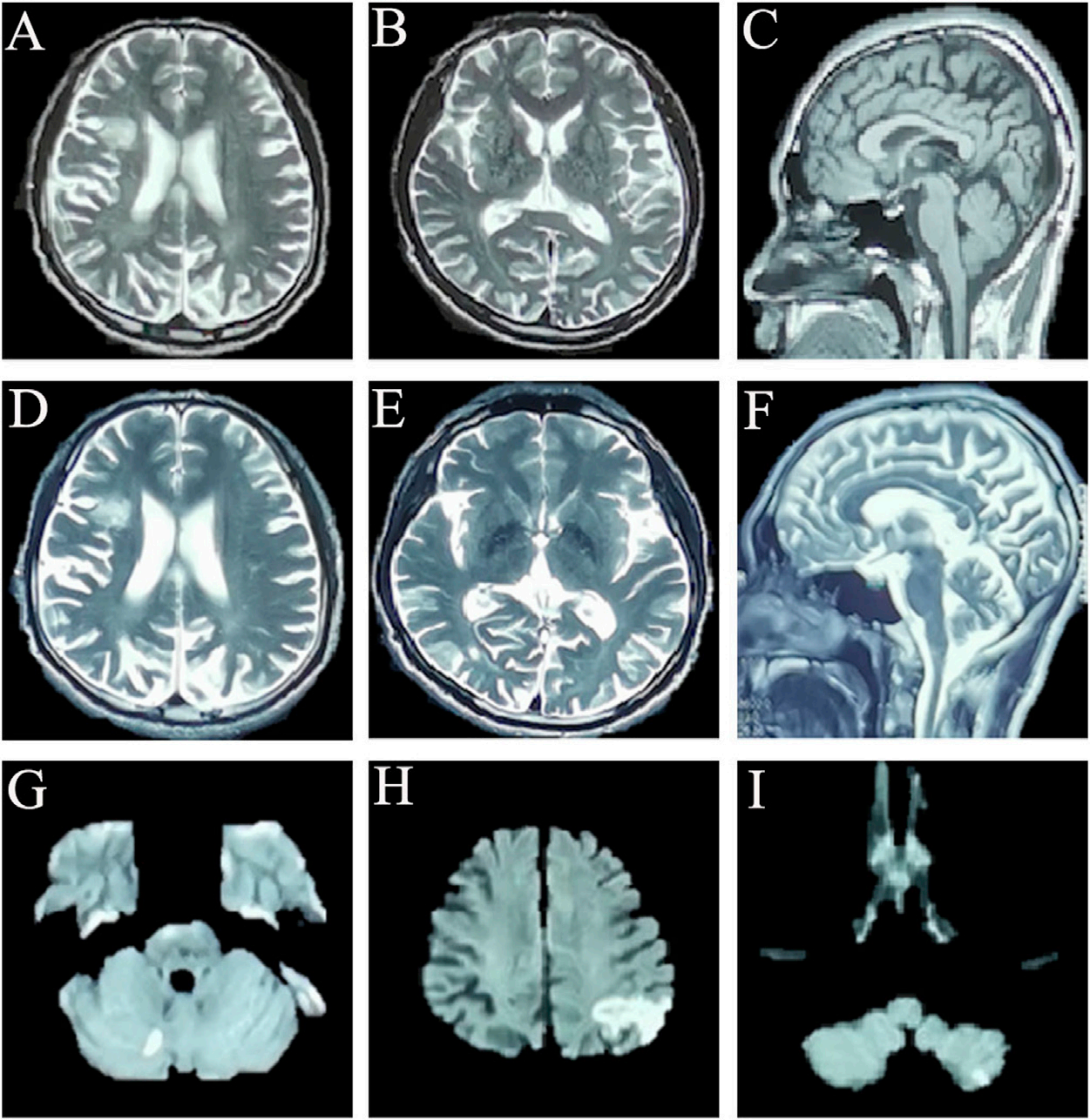
Cranial magnetic resonance imaging of this patient: **(A–C)** cross-sectional sequences of T2-weighted image (T2WI) **(A,B)** and sagittal image of T1-weighted image (T1WI) **(C)** (17 December 2019) showed the encephalomalacia in the right basal ganglia, frontal lobe and cerebral atrophy. **(D–F)** Cross-sectional sequences and sagittal images of T2WI (31 August 2021) showed that encephalomalacia foci had expanded to bilateral frontal lobe, right insula, and right temporo-occipital junction; brain atrophy is more worse than before. **(G)** Diffusion-weighted imaging (DWI) sequence showed punctate hyperintensity singals on the right cerebellum (22 September 2021). **(H,I)** DWI sequences showed abnormally high signal intensity on left parietal-occipital junction, and the left cerebellum (10 October 2021).

The patient is a 33-year-old male, an automobile factory worker with a bachelor’s degree, who presented to cognitive impairment clinic on 15 September 2021 with the chief compliant of “memory declines for 3 years and progressive deterioration for 5 months”. Three years before admission, the patient developed memory decline without obvious inducement, mainly in recent memory impairment. He was found to have reduced responsiveness during communication and sometimes was reluctant to communicate with others, occasionally incoherent, but he was still able to fulfill his job. His family noticed that he was emotionally indifferent, did not concerned about his family and family affairs as much as he had been in the past, and was emotionally depressed. Two years ago, the patient had visited the neurology outpatient department because of the above symptoms and underwent cranial magnetic resonance imaging (MRI) examination on 17 December 2019, which showed encephalomalacia in the right basal ganglia, frontal lobe and cerebral atrophy (Figures 1A–C). Five months ago, the patient felt that his memory loss was worsening, manifested by an inability to recall the events of the previous day, as well as to remember newly learned lyrics and melodies (he used to be good at music), and occasional transient difficulty typing on his mobile phones. Seven days prior to admission, the MRI (31 August 2021) results showed that the encephalomalacia foci had expanded to bilateral frontal lobe, right insula, and right temporo-occipital junction as compared to previous findings (Figures 1D–F). The pressure of cerebrospinal fluid (CSF) by lumbar puncture was normal, the CSF appearance was colorless and transparent, the routine examination was normal, and the biochemical and immune examinations were normal. His symptoms were not significantly improved after treatment with nutritional nerve and intellectual promotion, and he was later transferred to our cognitive impairment clinic and admitted to the neurology department. He had no significant medical history, denying arterial hypertension, diabetes, coronary heart disease (CHD), cerebrovascular disease, and other chronic diseases. Denied any history of infectious diseases such as hepatitis, tuberculosis and malaria; history of trauma and blood transfusion; history of food and drug allergy. He had been depressed after suffering an emotional trauma in 2011. A history of dog bite and rabies vaccination in 2018. Smoking history of 10 years, 20 cigarettes/day; no history of alcohol consumption. His uncle died and began to show signs of memory impairment at the age of 50. Denied any other family history of genetic problems. Physical examination: T 36.5°C, P 52 times/min, R 17 times/min, BP 127/87 mmHg (1 mmHg = 0.133 kPa), no deformity in the chest, lungs breath sounds clear, no wet and dry rales. The heart sounds were strong and rhythmic, and there were no pathological murmurs in the auscultation area of each valve. The abdomen was soft, without tenderness, and the liver and spleen cannot be palpable under the ribs. There was no swelling in the lower limbs, and no deformity of the spinal limbs. Neurological examination: clear in consciousness, fluent in language, time, place, character orientation are normal, calculation is normal, declined in recent memory, bilateral pupils equal in size, left: right for 3 mm:3 mm, pupillary light reflex was present, central eye position, ocular motility was normal, no diplopia or nystagmus, bilateral nasolabial sulcus were symmetrical, central tongue extension, soft palate elevation were normal, pharyngeal reflex (+), limbs muscle strength grade V, normal muscle tone, tendon reflex (++), bilateral Babinski sign (−), bilateral symmetric sensory examination, bilateral limbs ataxia examination were stable and accurate. The frontal lobe release signs of palm-grasping reflex, rooting reflex and sucking reflex were all negative.

### 2.2 Auxiliary examinations

The routine blood, coagulation function, serum immune function, liver and kidney function, lipids, blood glucose, high sensitive C-reactive protein, homocysteine, thyroid function, folic acid, vitamin B12, ferritin, immune panel (hepatitis A, hepatitis B, hepatitis C, syphilis and HIV), urine routine and stool routine showed no significant abnormalities. The electrocardiogram showed sinus rhythm and normal. No arrhythmias were detected by ambulatory electrocardiography (AECG). Cardiac color doppler ultrasound showed mild regurgitation of aortic valve, mitral valve and tricuspid valve. No intracardiac right-to-left shunts was detected by right heart contrast echocardiography. Color doppler ultrasound of abdomen and urinary system showed no abnormalities. Brain MRI (22 September 2021) results showed punctate hyperintensity on diffusion-weighted imaging (DWI) of the right cerebellum, suggesting an acute cerebral infarction, cerebral atrophy and encephalomalacia foci in different degree (Figure 1G). Brain magnetic resonance angiography (MRA) (22 September 2021) showed no significant abnormalities in the vascular morphology and structure (data not shown). The neuropsychological assessment showed multidomain cognitive decline, highlighted by delayed recall deficits, as well as emotional apathy and sleep disturbances (Table 1). The Dr. Brain tool was used to automatically perform brain tissue segmentation and extract multiparameter volumetric measurements of different brain regions for analysis (https://cloud.drbrain.net, registration number: 20212210359). The artificial intelligence brain structure imaging analysis showed that the proportion of gray matter and white matter to intracranial volume decreased, and the proportion of bilateral thalamus, caudate nucleus, putamen, bilateral frontal lobe, parietal lobe, occipital lobe, and temporal lobe decreased, especially reduced in the cerebellum regions (Table 2).

**TABLE 1:** Neuropsychological scale outcomes for the first visit and follow-up.

Neuropsychological test	First visit	3 Month	12 Month
MoCA (scores/subitems)	**18** (delayed recall, attention, orientation, language, visuospatial orientation and executive function)	**17** (delayed recall, attention, orientation, language, visuospatial orientation and executive function)	**19** (delayed recall, attention, orientation, language, visuospatial orientation and executive function)
MMSE (scores/subitems)	**26** (orientation, attention and calculation, delayed recall, structure)	**23** (orientation, writing, attention and calculation, delayed recall, structure)	**20** (orientation, writing, attention and calculation, delayed recall, structure)
ADAS-cog (scores/subitems)	**24** (word recall, word recognition, attention, structure, word finding difficulty)	**25** (word recall, word recognition, attention, structure, word finding difficulty)	**22** (word recall, word recognition, attention, structure, word finding difficulty)
CDR (scores)	**0.5**	**0.5**	**0.5**
BNT (scores)	**15**	**15**	**15**
ADL (scores/subitems)	**20**	**20**	**20**
NPI (scores/subitems)	**1** (apathy)	**1** (apathy)	**1** (apathy)
HAMD-21 (scores/subitems)	**5** (reduced ability to work, anxiety, sleep disturbances, retardation)	**5** (reduced ability to work, anxiety, sleep disturbances, retardation)	**5** (reduced ability to work, anxiety, sleep disturbances, retardation)

MMSE, mini-mental state examination; MoCA, montreal cognitive assessment; ADAS-cog, Alzheimer’s disease assessment scal-cognitive section; CDR, clinical dementia rating; BNT, Boston naming test; ADL, activity of daily life; NPI, neuropsychiatric inventory questionnaire; HADM-21, The 21-items Hamilton depression rating scale.

The bold values indicate the scores obtained by the patient in different cognitive areas, and we have included the explanations in the table where appropriate.

**TABLE 2:** The structural MRI features based on artificial intelligence analysis.

	Structure	Volume (cm^3^)	Ratio to intracranial volume (%)	Normal range (%)
The whole brain information	Gray matter	565.45	32.75%↓	39.48%–47.77%
	White matter	525.44	30.43%↓	32.46%–41.13%
	Total cerebrospinal fluid	635.60	36.81%	11.41%–26.88%
	Total intracranial space	1726.48	——	——
Thalamus	Left thalamus	2.23	0.13%↓	0.22%–0.42%
	Right thalamus	2.45	0.14%↓	0.23%–0.42%
Caudate nucleus	Left caudate nucleus	2.02	0.12%↓	0.14%–0.22%
	Right caudate nucleus	1.24	0.07%↓	0.15%–0.23%
Putamen	Left putamen	2.67	0.15%↓	0.18%–0.31%
	Right putamen	1.78	0.10%↓	0.18%–0.30%
Frontal lobe	Left frontal lobe	44.29	2.57%↓	2.84%–3.98%
	Right frontal lobe	43.76	2.53%↓	2.83%–4.24%
	Left superior frontal gyrus	12.30	0.71%↓	0.74%–1.06%
	Right superior frontal gyrus	10.16	0.59%↓	0.70%–1.08%
	Left middle frontal gyrus	13.06	0.76%↓	1.05%–1.41%
	Right middle frontal gyrus	15.10	0.87%↓	1.03%–1.39%
	Left inferior frontal gyrus	2.43	0.14%↓	0.17%–0.27%
	Right inferior frontal gyrus	2.14	0.12%↓	0.18%–0.29%
Parietal lobe	Left parietal lobe	44.29	2.57%↓	2.84%–3.98%
	Right parietal lobe	42.38	2.45%↓	2.86%–4.34%
	Left supramarginal gyrus	7.89	0.46%	0.39%–0.66%
	Right supramarginal gyrus	5.40	0.31%↓	0.39%–0.59%
	Left precuneus	9.30	0.54%↓	0.60%–0.88%
	Right precuneus	9.74	0.56%↓	0.64%–0.90%
	Left angular gyrus	7.60	0.44%↓	0.53%–0.74%
	Right angular gyrus	10.82	0.63%↓	0.64%–0.89%
Occipital lobe	Left occipital lobe	27.01	1.56%↓	1.98%–2.98%
	Right occipital lobe	30.42	1.76%↓	2.04%–3.02%
Temporal lobe	Left temporal lobe	39.77	2.30%	2.23%–3.11%
	Right temporal lobe	39.78	2.30%↓	2.36%–3.20%
Insula	Left posterior insula	2.46	0.14%	0.12%–0.18%
	Right posterior insula	2.31	0.13%↓	0.15%–0.20%
Cingulate gyrus	Left cingulate gyrus	11.28	0.65%↓	0.75%–1.07%
	Left anterior cingulate gyrus	4.39	0.25%↓	0.30%–0.39%
	Left middle cingulate gyrus	3.26	0.19%↓	0.23%–0.35%
	Left posterior cingulate gyrus	3.63	0.21%↓	0.22%–0.33%
Cerebellum	Left cerebellum cortex	36.71	2.13%↓	2.32%–3.23%
	Total cerebellum volume	99.28	5.75%↓	5.84%–8.30%

**FIGURE 2:**
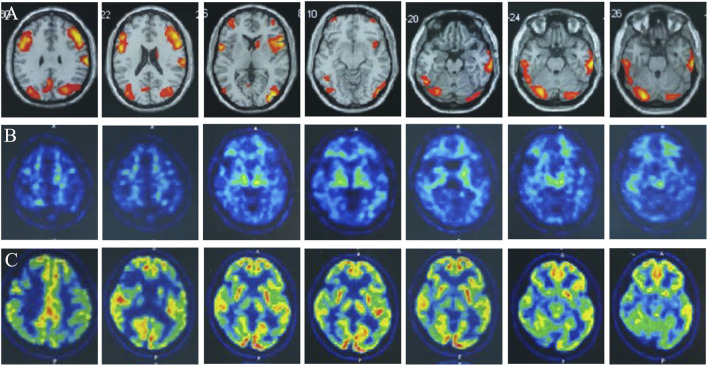
Multimodality molecular imaging of the patient: **(A)** Multimodal molecular imaging of 18-fluoro-2-deoxy-D-glucose positron emission tomography (^18^F-FDG PET-CT) results suggested hypometabolism in dorsolateral frontal cortex, dorsolateral parietal cortex, bilateral precuneus, lateral of right temporal lobe and right occipital lobe, and right caudate nucleus. **(B)** 11-carbon-Pittsburgh positron emission tomography (^11^C-PIB PET-CT) showed no amyloid plaque deposition in the brain. **(C)** 18-fluoro-S16-positron emission tomography (^18^F-Flortaucipir-tau PET-CT) showed tau deposition in bilateral thalamus, caudate nucleus, midbrain, pons, and bilateral medial temporal lobe.

**FIGURE 3:**
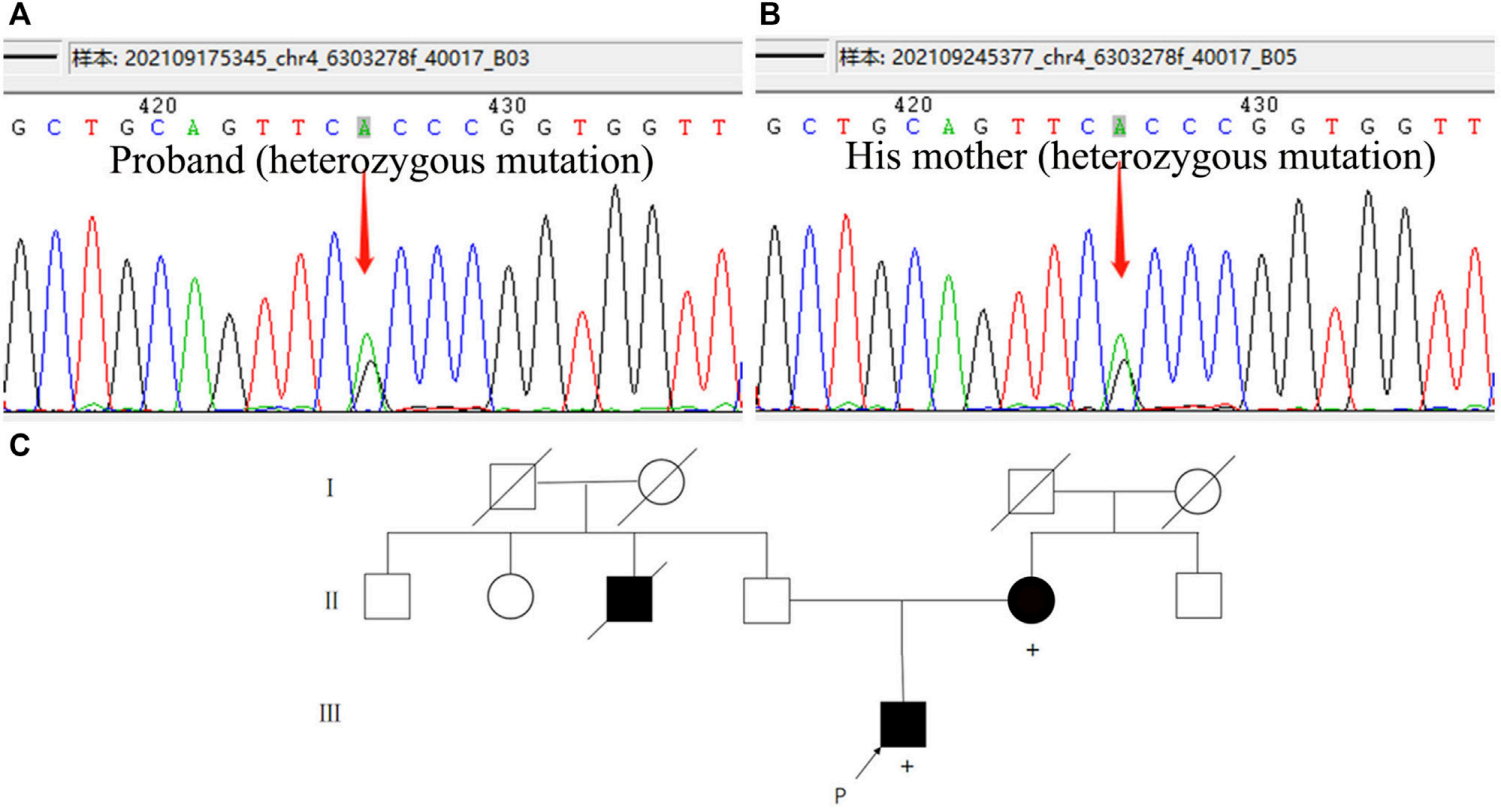
Whole exome sequencing results and family pedigree. **(A,B)** Both of the patient and his mother carried a heterozygous mutation in *WFS1* c.1756G>A p.A586T. **(C)** Genealogical tree of the patient. [Square, man; circle, woman; black-filled symbol, affected individual (with cognitive impairment); empty symbol, unaffected individual; P (arrow), proband; diagonal black line, deceased; +, individual with *WFS1* gene mutation; consanguineous marriage was not observed in this family].

We performed lumbar puncture and CSF examination for the patient again. The CSF pressure was 133 mm H_2_O, colorless and transparent; routine: total cell count was 8 × 10^6^/L, white blood cell count was 2 × 10^6^/L (normal value: 0–8); biochemistry: chloride level was 126 mmol/L (normal values: 120–132 mmol/L), glucose level was 3.46 mmol/L (normal value: 2.5–4.5 mmol/L), lactate level was 1.3 mmol/L (normal value: 0.6–2.2 mmol/L), protein level was 0.27 g/L (normal value: 0.15–0.45 g/L), adenosine dehydrogenase was 0.03 U/L (normal value: 0–8 U/L); Gram, acid-fast and ink staining showed no bacteria. Immunoglobulin G level was 8 mg/L (normal value: 0.1–34 mg/L), immunoglobulin A level was 7.25 mg/L (normal value: 0.1–5 mg/L)↑, immunoglobulin M level was 0.8 mg/L (normal value: 0–1.3 mg/L), and the albumin level was 120 mg/L (normal value: 0–350 mg/L). Oligoclonal band, central nervous system demyelinating disease spectrum antibody (AOP4; MBP; MOG; Flotillin-1/2), anti-lgLon antibody detections were negative. CSF Neurofilament light (Nfl) level was 9291.08 pg/mL↑ (normal value: 30–39 years old, <380 pg/mL), beta-amyloid (1–42) (Aβ_1-42_) level was 837.34 pg/mL (normal value: >651 pg/mL), beta-amyloid (1–40) (Aβ_1-40_) level was 4171.40 pg/mL, Aβ_1-42_/Aβ_1-40_ ratio was 0.2 (normal value: >0.1), phosphorylated tau protein (p-tau_181_) was 15.80 pg/mL (normal value: ≤61 pg/mL), and total tau protein was 335.85 pg/mL (normal value: <290 pg/mL)↑. Multimodal molecular imaging of 18-fluoro-2-deoxy-D-glucose positron emission tomography (^18^F-FDG PET-CT) results suggested hypometabolism in dorsolateral frontal cortex, dorsolateral parietal cortex, bilateral precuneus, lateral of right temporal lobe and right occipital lobe, and right caudate nucleus (Figure 2A). 11-carbon-Pittsburgh positron emission tomography (^11^C-PIB PET-CT) showed no amyloid plaque deposition in the brain (Figure 2B). 18-fluoro-S16-positron emission tomography (^18^F-Flortaucipir-tau PET-CT) showed Tau deposition in bilateral thalamus, caudate nucleus, midbrain, pons, and bilateral medial temporal lobe (Figure 2C). The whole exome gene sequencing results of the patient and her mother showed that both of them carried a heterozygous missense mutation in WFS1 c.1756G>A (p.A586T) (Figures 3A, B). Therefore, we performed a detailed detailed family history questioning and screening, the patient’s mother had clinical manifestations of cognitive decline and emotional anxiety, which were confirmed by the objective neuropsychological scales (the MMSE score is 25, MoCA score is 23 and NPI score is 2). His uncle was described by his family to memory loss and gradually progressed to the inability to recognize his family members, accompanied by abnormal behavior symptoms, however, no formal medical treatment was received until his death at the age of sixty-seven. His grandparents were dead. His father and other blood relatives are currently in good health with no complaints of memory loss or clinical manifestations (Figure 3C).

### 2.3 Treatment and follow-up

The patient was temporarily treated with aspirin 100 mg qd, rosuvastatin 10 mg qn, and donepezil 5 mg qn for the first diagnosis of acute cerebral infarction and mild cognitive impairment. However, 1 month later, the patient returned to our hospital with the symptoms of “inability to calculate and write for 10 days,” presenting with further deterioration of memory impairment and typical Gerstmann syndrome. The brain MRI (10 October 2021) showed abnormally high signal intensity in the DWI sequence of the left parietal-occipital junction, and left cerebellar hemisphere, suggesting a new acute cerebral infarction (Figures 1H, I). Ophthalmic examination showed that the corrected visual acuity of the right eye was 0.12 and the left eye was 0.1, bilateral intraocular pressures (IOP) were 17 mmHg, and bilateral corneas were transparent. Optical coherence tomography (OCT) showed clear optic disc boundaries, no optic atrophy, and no significant abnormalities in the macula of both eyes. The patient failed to cooperate to complete the perimetry. The patient had no symptoms of hearing loss or tinnitus. The levels of various tumor markers, sex hormones, comprehensive analysis of organic acids and amino acids for genetic metabolic disease in urine, and acylcarnitine spectrum analysis were all normal. The level of neurospecific protein (S100β) was 123.42 pg/mL (0.05–0.18 ng/mL). Vasculitis related immunoantibodies, rheumatic immunoantibodies, antinuclear antibodies were negative. Lactate exercise test results were negative [morning lactate level was 3.27 mmol/L (normal value:0.4–2 mmol/L), and half an hour after exercise lactate level was 2.69 mmol/L (normal value:0.4–2 mmol/L)]. During the subsequent follow-up, the patient again had another insidious ischemic stroke event, but without significant changes in clinical symptoms.

## 3 Discussion

Autosomal recessive inheritance of the *WFS1* gene always lead to classic WS1, a rare and severe disease characterized by juvenile-onset diabetes, progressive neurologic degeneration, and endocrine dysfunction (Li et al., 2020). However, only 14%–58% of patients present with the four typical clinical manifestations of diabetes insipidus, diabetes mellitus, optic atrophy and deafness, which is called complete WS1 (Barrett et al., 1995; Medlej et al., 2004). In addition, recent literature reports have proposed that autosomal dominant mutations have been described as responsible for WS-like disease (Eiberg et al., 2006; Rendtorff et al., 2011). Their common features are mostly attributed to the incomplete or a predicted partial loss of functional mutations that disrupt wolframin protein (Pallotta et al., 2019). In this report, we described a young male patient with a particular clinical manifestations of cognitive impairment and recurrent stroke. Genome-wide sequencing revealed a heterozygous missense mutation in exon 8 of the *WFS1* gene (c.1756G>A p.A586T). Although his clinical symptoms were insufficient to make the diagnosis of WS1, a detailed screening of multimodal molecular imaging, CSF biomarkers, and genetic analysis consistently suggested a role for Wolframin in the disease. Therefore, we hypothesized that this novel mutation could serve as an etiological basis for explaining the patient’s neurodegenerative damage and susceptibility to ischemic injury.

The human *WFS1* gene maps to chromosome 4p16, and is composed of eight exons. Among the eight exons of *WFS1* gene, exon 8 has the most clinical significance and a majority of the identified mutations of *WFS1* occur in this largest exon (Inoue et al., 1998; Strom et al., 1998). The *WFS1* gene encodes an 890 amino acid Wolframin transmembrane protein, which is highly expressed in the hippocampus, amygdala, allocortex, and olfactory bulb (Takeda et al., 2001), pancreas and the heart (Fonseca et al., 2010). The important physiological function of Wolframin protein is attributed to its localization in the ER, and as a UPR component, it promotes cell survival by mitigating ER stress signaling. The deficiency of wolframin protein caused by mutations in the *WFS1* gene will lead to insufficient activation of the UPR to cope with the accumulation of unfolded proteins within the ER lumen, which inevitably leads to apoptosis (Harding and Ron, 2002). This pathogenic mechanisms lay the foundation of progressive neurodegeneration and endocrine dysfunction in WS1. Different mutation forms of *WFS1* gene (missense, frameshift, or deletion) will lead to different types of wolframin alterations. Wolframin dysfunction can be manifested as predicted complete, partial, or putative minor loss, resulting in heterogeneity of clinical presentation (Rohayem et al., 2011). We identified a novel *WFS1* missense mutation p.A586T, c.1756 G→A in exon 8. This mutation changes the transmembrane region of the protein by substitution of the non-polar, hydrophobic amino acid alanine for the polar, hydrophilic amino acids threonine, which is putatived to alter the structural portion of Wolframin protein located within the lumen of the ER. This form of mutation underlies the genetic pathogenic basis for the central nervous system injury caused by Wolframin protein in this patient. The cognitive decline and mood disorders exhibited by her mother, as well as the carriage of the mutation at the same locus, also further validate our speculation on the pathogenesis of the disease. It is difficult to establish a clear genotype-phenotype correlations due to the molecular complexity Wolframin protein, low prevalence and absence of a WS1 register (Pallotta et al., 2019). The sequence of occurrence of clinical features and the natural histories of WS1 have always been different. In addition to the four typical features, nearly 62% of WS1 patients will develop a wide range of neurological and psychiatric complications, including cerebellar ataxia, cognitive decline depression and anxiety (Li et al., 2020). Respiratory failure or dysphagia due to neurodegenerative damage to the brain stem are the common causes of mortality (Barrett et al., 1995). What is very interesting about this case is that the patient presented with cognitive impairment as the initial symptom and had recurrent ischemic stroke events during we searched for the cause of cognitive decline. Although no clear pathogenic mechanism has been established between Wolframin protein loss and neuronal ischemic injury, some case reports and researches have provided good hints and possible explanations for its pathogenesis. It has been confirmed that Wolframin protein is highly enriched in the central extended amygdala, ventral striatum, hippocampal CA1 region, prefrontal cortex and central auditory pathway by mouse model. This specific distribution of neuroanatomy suggests that Wolframin dysfunction may contribute to the neurological and psychiatric symptoms found in WS1 (Luuk et al., 2008). Neurons in these brain regions with prominent Wolframin expression are typically vulnerable to ischemia induced damage once Wolframin signal is spared or absent. This selective vulnerability might be related to the inhibition of protein synthesis promoted by Wolframin during ER stress induced by ischemia (Luuk et al., 2008; Schmidt-Kastner, 2015). However, neurons where Wolframin signal is absent are preserved during ischemic events (Li et al., 2020). We have screened a large number of young stroke etiologies, but there was no clear evidence of atherosclerosis, cardioembolism, immune and metabolism-related diseases, or arteritis etc. The ER stress response caused by Wolframin dysfunction is speculated to be an important mechanism involved in ischemic injury in this patient, nevertheless, further mechanistic studies are needed to confirm.

The study of Chaussenot et al. (2011) found that cognitive impairment was ranked the third (32%) among the neurologic complication of 59 WS1 patients, only second to cerebellar ataxia and peripheral neuropathy. Studies have confirmed that almost all patients with WS1exhibit a decrease in brainstem and cerebellar volume, regardless of age, duration of diabetes, or other disease characteristics. In addition, decreased cortical thickness was also observed in areas localized in WS1, including lingual, precentral, and rostral middle frontal cortex, which are associated with vision, motor function and cortical functions such as working memory, respectively. These data together suggest that the effects of Wolframin protein on brain development are both globally and preferentially, even at the earliest stages of symptoms (Hershey et al., 2012). The Artificial intelligence brain structure imaging analysis showed that the patient developed diffuse cerebral atrophy at a young age, especially in the cerebellum, with cortical and subcortical regions involving bilateral thalamus, caudate nucleus, putamen, bilateral frontal lobe, parietal lobe, occipital lobe and temporal lobe. The changes of brain structure and brain atrophy location were consistent with previous studies. Despite the diffuse atrophy of brain structures, the pattern of cognitive impairment in patients with WS differs from that of typical Alzheimer’s disease (AD). The effects of WFS1 functional deficits on cognitive and mood responses manifest as a complex behavioral pattern with some gender-related differences in impairment intensity. In mice with a complete knockout of exon 8 of the *WFS1* gene (*Wfs1* ^*△*Exon8^), memory deficits were very consistent in males and observable in all the procedures tested, particularly marked for spontaneous alternation and novel object tests. However, the spatial information of the platform position measured during the water maze detection test was not affected. This suggested an incomplete impairment of spatial and non-spatial, short-term and long-term memory (Crouzier et al., 2022). Neurodegenerative changes caused by Wolframin protein dysfunction share common pathophysiological signaling pathways with AD, including the pathways related to Ca^2+^ signaling dysregulation, ER stress and UPR, impaired mitochondrial function, autophagic pathology, and excitotoxicity. It has been demonstrated in fly models that neuronal knockdown of *WFS1* exacerbated axonal degeneration induced by overexpression of human tau (Sakakibara et al., 2018), and it is presumed that pathologic tau will be propagated along the WFS1^+^ entorhinal cortex layer II (ECII)—hippocampal CA1 pathway, inducing disconnection in this circuit, contributing to the memory deficits observed in early AD stages and accelerating neurodegenerative damage (Delpech et al., 2021). Recently, Chen et al. (2022) has reported that WFS1 deficiency is associated with increased tau pathology and neurodegeneration, whereas overexpression of WFS1 reduces those changes. They found that overexpression of WFS1 can reverse changes in levels of ER stress and autophagy lysosomal pathway (ALP)-related proteins in WFS1-deficient tau transgenic mice, suggesting a possible role for WFS1 in the regulation of tau pathology and neurodegeneration through chronic ER stress and the downstream ALP. These data indicate a novel role for Wolframin in neuronal susceptibility to neurodegeneration-related tau pathology. The PET-CT of this patient showed tau deposition in both cortical and subcortical structures. However, the causal relationship and interaction between tau pathological changes and *WSF1* gene mutation still need to be further explored and confirmed.

In conclusion, our data report a case of a young male patient with early cognitive impairment as his initially prominent manifestation and recurrent cerebral infarction. Although we did not identify the direct etiology of his recurrent ischemic strokes, the neurological damage caused by *WFS1* missense mutation may become our speculation on the pathogenesis of this disease. Our findings illustrated that *WFS1* mutations could be associated with a broader range of clinical phenotypes than previously reported. The clinical features of diseases associated with *WFS1* mutations are highly heterogeneous, and some phenotypes are not fully understood, even for the same locus mutation, the phenotype varies among individuals. Through a meticulous description of the clinical characterization of mutations in this gene will help us to obtain greater insights into the normal biological functions of Wolframin protein*,* as well as other candidate genes involved in cognitive disorders and neurodegenerative diseases. Subsequent follow-up of this patient and his family, monitoring of the disease progression, and the construction of an animal model of mutation at this locu to confirm its pathogenicity are necessary.

## Data Availability

The datasets presented in this study can be found in online repositories. The names of the repository/repositories and accession number(s) can be found in the article/supplementary material.
